# Urinary Melatonin Levels and Skin Malignancy

**Published:** 2014-01

**Authors:** Reza Ghaderi, Samineh Sehatbakhsh, Mehdi Bakhshaee, Gholam Reza Sharifzadeh

**Affiliations:** 1 Department of Dermatology, Birjand University of Medical Sciences, Birjand, Iran;; 2 The Sinus and Endoscopic Surgery Research Center, Mashhad University of Medical Sciences, Mashhad, Mashhad, Iran;; 3 Department of Epidemiology, Birjand University of Medical Sciences, Birjand, Iran

**Keywords:** Melatonin, Skin neoplasm, Basal cell carcinoma, Squamous cell carcinoma

## Abstract

Melatonin inhibits tumor genesis in a variety of in vivo and in vitro experimental models of neoplasia. In industrialized societies, light at night, by suppressing melatonin production, poses a new risk for the development of a variety of cancers such as breast cancer. This effect on skin has been previously studied only in animals and not in humans. Our goal was to examine the relationship between 24-hour 6-sulphatoxymelatonin levels and skin cancer in a case-control study of 70 patients with skin cancer and 70 healthy individuals. The level of 6-sulfatoxymelatonin was measured in 24-hour urine by the ELISA method. In the case group, 55 (78%) patients had basal cell carcinoma and 15 (22%) had squamous cell carcinoma. The mean level of 24-hour urine 6-sulfatoxymelatonin was significantly higher in the control group (P<0.001). Also, sleep duration had a significant difference between the two groups (P=0.001). It seems that a low level of 24-hour urinary 6-sulfatoxymelatonin renders human beings prone to skin cancer. This association, however, requires further investigation.

## Introduction

Melatonin inhibits tumor genesis in different experiments, both in vivo and in vitro. Studies regarding melatonin and cancers show that melatonin exerts its anti-cancer effect via three mechanisms: inhibition of cell proliferation, stimulation of differentiation, and apoptosis.^1^^,^^2^

Melatonin’s effects as an antioxidant include: a) cleaning free radicals; b) increasing antioxidative enzymes; c) stimulating mitochondrial oxidative phosphorylation and decreasing electron leakage; and d) stimulating other antioxidant effects.^3^

There is an assumption that in developed societies, light exposure at night increases the risk of breast cancer and some other cancers by suppressing melatonin.^1^^,^^2^ Considering the effects of melatonin in the treatment of breast cancer^4^^,^^5^ and prostate cancer,^6^ we can posit that it can be helpful in the prevention or treatment of skin cancer [basal cell carcinoma (BCC) and squamous cell carcinoma (SCC)].

Experimentally it has been shown that melatonin plays some roles in skin physiology such as hair growth cycling, fur pigmentation, and melanoma control. Melatonin suppresses ultraviolet (UV)-induced damage to skin cells and exerts strong antioxidant effects on UV-exposed cells.^7^

Melatonin is transformed to 6-hydroxymelatonin and N^ 1 ^–acetyl-N^ 2 ^–formyl-5-methoxy-kynuramine in melanocytes, keratinocytes, and fibroblasts primarily. All three types of cells in the skin express the metabolism of melatonin and its endogenous production.^8^

Based on animal studies, the use of melatonin, Metformin, and their combination causes a significant reduction in the number and size of skin tumors in the mice with benzo(a)pyrene solution on their skin.^9^

Although there are several studies on the effect of melatonin on different human cancers, including breast,^4^^,^^10^^-^^12^ prostate,^6^ colorectal,^13^and endometrial cancers,^2^ such possible effect on human skin cancer has yet to be investigated. Therefore, given the melatonin effect on the skin,^13^^,^^14^ in a novel approach we investigated the association between the 24-hour urinary 6-sulfatoxymelatonin level and skin cancer in human beings (BCC and SCC).

## Materials and Methods

This case-control study recruited 140 people, including 70 patients with skin cancer (confirmed by pathologists) and 70 healthy individuals. The sample size was estimated to be 70 persons in each group, based on Altman’s nomogram and the results of previous studies (SD=0.67 and power=0.8). The 24-hour urinary melatonin metabolite (6-sulphatoxymelatonin) level was measured by the ELISA method. Outcome measures were age, sex, sleep duration, job, related drugs, smoking, and sun and light exposure duration. Sleep quality was classified according to 12 standard questions, adopted from a sleep history questionnaire^15^ (table 1). The questionnaire was first translated into Farsi, i.e. the language of the study population, and then converted into English. Score 1 was allocated to each question if the answer was yes, scores between 0 and 3 denoted sleeping well (first group), scores between 3 and 6 indicated sleeping quite well (second group), scores between 6 and 9 signified sleeping quite badly (third group), and scores between 9 and 12 stood for sleeping badly (fourth group). Regarding the reliability and validity of the questionnaire, after completing the first 35 questionnaires, based on SPSS, Cronbach’s alpha coefficient was reported to be 0.7526 (>0.7). Accordingly, the questionnaire had reasonable validity and reliability in this study.

**Table 1:** Authors’ revised sleep history questionnaire (adopted from tenth edition of Kaplan & Sadock synopsis of psychiatry)

		**Yes (Score 1)**	**No (Score 0)**
1	Do you feel sleepy or have sleep attacks during the day?		
2	Do you nap during the day?		
3	Do you have trouble concentrating during the day?		
4	Do you have trouble falling asleep when you first go to bed?		
5	Do you awaken during the night?		
6	Do you awake more than once?		
7	Do you awaken too early in the morning?		
8	Do others live at home who interrupt your sleep?		
9	Are you regularly awakened at night by pain or the need to use the bathroom?		
10	Does your job required shift changes or travel?		
11	What sleep medications, prescription or nonprescription, do you take?		
12	Have you ever suffered from depression, anxiety, or similar problems?		

This study was approved by the Ethics Committee of Birjand University of Medical Sciences. All the patients and controls were fully informed about the study protocol, and a signed informed consent was obtained from each of them.

The data were then analyzed with SPSS software (version 13). Age and sleep requirement between the two groups were compared using the *t* test. Also, the Kruskal–Wallis test was used to compare the mean level of 24-hour urine 6-sulfatoxymelatonin (in hours) in each group, and the Mann-Whitney test was employed to compare the mean level of 24-hour urine 6-sulfatoxymelatonin between the two groups. Additionally, the chi-squared test was utilized to compare sleep distribution from the point of quality and quantity between the two groups. A p value less than 0.05 was considered statistically significant.

## Results

The study population comprised 140 individuals, divided into two equally numbered groups: a case group at a mean age of 54.8±12.2 and a control group at a mean age of 54.4±12.1 years. Age and sex between the two groups were comparable (table 2).

**Table 2:** Demographic data and melation in level of the patients compared with the controls

	**Cases**	**Controls**	**P value**
Age	54.8±12.2	54.4±12.1	0.86
Sex (Male/Female)	42.9%/57.1%	38.6%/61.4%	0.61
Melatonin level	15.9±8.1	47±23.6	<0.001

There was no significant difference between the case and control groups regarding sun and light exposure duration (P=0.9).

The mean level of 24-hour urine 6-sulfatoxymelatonin in the case group was 15.9±8.1, while it was 47.0±23.6 in the control group. This was in accordance with the duration (quantity) and sleep quality in the two groups inasmuch as the control group slept more and better than did the case group generally (P=0.005) (table 3).

**Table 3:** Sleep quantity and sleep quality of the patients compared with the controls

	**Cases**	**Controls**	**P value**
Sleep Quantity			
Less than 6 hours	20 (28.6)	8 (11.4)	
Between 6 and 8 hours	38 (54.3)	30 (42.9)	0.001
More than 8 hours	12 (17.1)	32 (45.7)	
Sleep Quality			
Good	21 (30)	37 (52.9)	
Almost good	20 (28.6)	21 (30)	0.005
Almost bad	23 (32.9)	7 (10)	
Bad	6 (8.5)	5 (7.1)	

## Discussion

The results of the present study demonstrated that the case group had a lower level of 24-hour urinary 6-sulfatoxymelatonin, which may be interpreted that lower levels of melatonin are correlated with a rise in the risk of SCC and BCC. Our results chime in with those in a similar study on breast cancer, which calculated 6-sulphatoxymelatonin levels in urine.^10^

Based on the results of our study, the average nocturnal sleeping hours was higher in the control group and it had a direct association with the amount of urinary melatonin (P<0.01). Meanwhile, in a similar study on breast cancer, the incidence of breast cancer had an inverse association with daily sleeping hours.^11^

Another similar study investigated the association between night-shift work and endometrial cancer. The risk of endometrial cancer had an upward trend in people who had rotating night shifts and obesity. However, the results of our study showed that the control group was more obese than was the case group.^2^

In an animal study conducted on 200 mice divided into 4 groups, benzo(a)pyrene solution was applied onto a skin site for 26 weeks. Melatonin, Metformin, or both were used in the animals in a parallel way. This promoted a significant reduction in the number and size of skin tumors.^9^

In a study done on the related mechanisms of cancer, melatonin inhibited the proliferation of malignant cells in breast cancer and hepatoma. Also, melatonin was reported to be an oncostatic agent via its augmentation on natural killer (NK) cells.^14^

To the best of our knowledge, the existing literature lacks any study on the probable impact of the melatonin level on predisposition to human skin cancer, although there are a few animal studies on the effect of melatonin in the prevention of skin carcinogenesis.^9^

## Conclusion

It seems that there is an association between the risk of skin cancer (SCC and BCC) and low levels of urinary 6-sulfatoxymelatonin, which is related indirectly to regular nocturnal sleep. This suggests that melatonin and regular nocturnal sleep may help prevent skin cancers.
